# The Cyclophilin-Dependent Calcineurin Inhibitor Voclosporin Inhibits SARS-CoV-2 Replication in Cell Culture

**DOI:** 10.3389/ti.2022.10369

**Published:** 2022-06-24

**Authors:** Natacha S. Ogando, Erik Metscher, Dirk Jan A. R. Moes, Eline J. Arends, Ali Tas, Jennifer Cross, Eric J. Snijder, Y. K. Onno Teng, Aiko P. J. de Vries, Martijn J. van Hemert

**Affiliations:** ^1^ Department of Medical Microbiology, Leiden University Medical Center, Leiden, Netherlands; ^2^ Department of Clinical Pharmacy and Toxicology, Leiden University Medical Center, Leiden, Netherlands; ^3^ Leiden Transplant Center, Leiden University Medical Center, Leiden, Netherlands; ^4^ Department of Nephrology, Leiden University Medical Center, Leiden, Netherlands; ^5^ Aurinia Pharmaceuticals Inc., Victoria, BC, Canada

**Keywords:** SARS-CoV-2, kidney transplantation, tacrolimus, voclosporin, cyclosporin A, calcineurin inhibitors

## Abstract

Kidney transplant recipients (KTRs) are at increased risk for a more severe course of COVID-19, due to their pre-existing comorbidity and immunosuppression. Consensus protocols recommend lowering immunosuppression in KTRs with severe acute respiratory syndrome coronavirus 2 (SARS-CoV-2) infection, but the optimal combination remains unclear. Calcineurin inhibitors (CNIs) are cornerstone immunosuppressants used in KTRs and some have been reported to possess antiviral activity against RNA viruses, including coronaviruses. Here, we evaluated the effect of the CNIs tacrolimus, cyclosporin A, and voclosporin (VCS), as well as other immunosuppressants, on SARS-CoV-2 replication in cell-based assays. Unexpected, loss of compound due to plastic binding and interference of excipients in pharmaceutical formulations (false-positive results) complicated the determination of EC50 values of cyclophilin-dependent CNI’s in our antiviral assays. Some issues could be circumvented by using exclusively glass lab ware with pure compounds. In these experiments, VCS reduced viral progeny yields in human Calu-3 cells at low micromolar concentrations and did so more effectively than cyclosporin A, tacrolimus or other immunosuppressants. Although, we cannot recommend a particular immunosuppressive regimen in KTRs with COVID-19, our data suggest a potential benefit of cyclophilin-dependent CNIs, in particular VCS in reducing viral progeny, which warrants further clinical evaluation in SARS-CoV-2-infected KTRs.

## Graphical Abstract

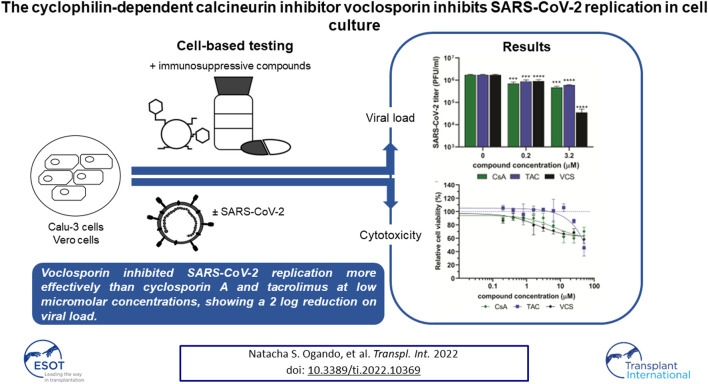

## Introduction

Between December 2019 and May 2022, severe acute respiratory syndrome coronavirus-2 (SARS-CoV-2), the causative agent of coronavirus disease-2019 (COVID-19), has resulted in over 500 million cases of infection with a reported estimated death toll of 6 million people globally (1). The severity of clinical manifestations of COVID-19 has been correlated to various comorbidities commonly present in transplant recipients (2–4). Moreover, some reports showed that transplant recipients are at increased risk of a more severe course of COVID-19 and related death (2–6).

Finding the right balance between preventing rejection and controlling infections is generally the conundrum when prescribing immunosuppression for transplant recipients (7). The current standard of care, specifically in kidney transplant recipients (KTR) consists of a calcineurin inhibitor (CNI), either tacrolimus (TAC) or cyclosporin A (CsA), an antimetabolite agent such as mycophenolate (MPA/MPS) and most often corticosteroids. An mTOR inhibitor such as everolimus (EVL) as part of the regimen may also be prescribed alternatively (8). So far, the precise impact of immunosuppression on the course of COVID-19 and the excess mortality observed in KTRs (9) is poorly understood. On the one hand, (over)immunosuppression might hamper antiviral responses to control SARS-CoV-2 infection, whereas unopposed (hyper-) inflammation from immune overactivation is thought to result in a more severe disease course. Consequently, consensus protocols recommend to reduce but not completely cede immunosuppression in SARS-CoV-2-infected KTR’s, depending on the risk of rejection and disease severity (10, 11).

Previous reports suggested that CNIs have antiviral activity against coronaviruses (12). TAC (which targets FKBP12) was reported to inhibit CoV replication in cell culture (13), and was recently proposed as a potential inhibitor of SARS-CoV-2 replication by computational analysis (14). Next to its immunosuppressive effects (15–20), CsA was reported to inhibit replication of different RNA viruses in cell culture (17, 21, 22), including human and zoonotic CoVs (20, 23–26). Several non-immunosuppressive CsA derivatives, like alisporivir (Debio-025), also inhibit the replication of CoVs in cell culture (15, 24, 27), including SARS-CoV-2 (28, 29). Collectively, these studies established the broad-spectrum antiviral activity of CsA and derivatives in cell culture-based infection models. These studies suggested that cyclophillins (cyps) are involved in CoV replication. However, knock-down of different Cyps in cells lead to variable effects on the replication of different CoVs (20, 24, 25, 30). Thus, the exact role of Cyps host proteins in CoV replication remains elusive (30, 31). Still, CsA has been suggested as the drug-of-choice for KTRs during the COVID-19 pandemic as an alternative to other regimens to prevent rejection (32).

Voclosporin (VCS) is a novel CNI which has been studied in psoriasis and renal organ transplantation. Additionally, VCS was recently FDA-approved for treatment of active lupus nephritis in combination with background immunosuppressive therapy (33–35). Structurally similar to CsA, VCS incorporates a methyl group at the amino acid residue position 1, which enhances its binding to calcineurin, and confers better metabolic stability (36, 37). (Pre)clinical observations suggested that VCS is more potent and less toxic at therapeutic levels than other immunosuppressants in its class, including CsA (34, 36–40). VCS was shown to inhibit norovirus replication in a CypA-dependent manner and more effectively than CsA (19). Therefore, VCS is an interesting candidate to evaluate for inhibitory activity on SARS-CoV-2 replication.

In this study, we compared the effect of three calcineurin inhibitors (TAC, CsA, VCS) and other immunosuppressants commonly used in transplant medicine on SARS-CoV-2 replication using cell-based assays. Our results showed that out of the three calcineurin inhibitors VCS was the most potent inhibitor of SARS-CoV-2 replication, using cell-based assays. Since VCS is also a more potent immunosuppressant than CsA with comparable potency to TAC, we concluded that VCS might be an interesting CNI to investigate further in KTRs COVID-19 patients.

## Material and Methods

### Virus and Cell Lines

For all infections, SARS-CoV-2 isolate Leiden-0002 (GenBank MT510999) was used (41). Vero E6 cells and Calu-3 2B4 cells (42), were cultured and infected as described previously (41). All experiments with infectious SARS-CoV-2 were performed in the LUMC biosafety level 3 facilities.

### Immunosuppressive Compounds

Voclosporin (Lupkynis™_,_ Aurinia Pharmaceuticals Inc.), cyclosporin A (Neoral^®^, Novartis), tacrolimus (Prograf^®^, Astellas), mycophenolate mofetil (CellCept^®^, Roche) or everolimus (Certican^®^, Novartis) stock solutions were prepared by dissolving the pharmaceutical formulation of these drugs in dimethyl sulfoxide (DMSO). Placebo capsules and pure VCS powder (Aurinia Pharmaceuticals Inc.), Tacrolimus (PHR1809), cyclosporin A (30024) and mycophenolic acid (M5255) (all from Sigma-Aldrich) and remdesivir (RDV; HY-104077, MedChemExpress) were dissolved in DMSO and stored at −20°C as single-use aliquots. Remdesivir was used as a standard positive control in all experiments.

### Measurement of Cyclosporin A, Tacrolimus and Voclosporin Concentrations by LC-MS/MS

Before analysis, samples were diluted in methanol and subsequently in blank whole blood to fall within the calibration line of 0-to 600 μg/L of VCS. Human whole blood was added to a final volume of 200 µL and mixed with 200 µL of 0.1 M zinc-sulphate and 500 uL of internal standard solution (32 ug/L of VCS D_4_ in acetonitrile). After centrifugation, supernatant was transferred to an autosampler vial after which 20 µL was injected into LC-MS/MS system. Quantification of VCS was performed with LC-MS/MS using a Thermo Quantiva UPLC-MS/MS system (ThermoFisher Scientific) (43), similarly to the validated protocol for measuring CsA and TAC. The equipment consisting of an Ultimate 3000 series UHPLC system, coupled to a TSQ Quantiva triple stage quadrupole mass spectrometer was used. Chromatographic separation was achieved using an Acquity UPLC BEH C18 1.7 µm; 2.1 × 50 mm column coupled to a VanGuard BEH C18 1.7 µm precolumn. Online solid phase extraction was performed using a Xbridge 10 µm 30 × 2.1 mm column. This protocol was validated according to the EMA bioanalytical method validation guideline (44).

### Cytopathic Effect Reduction Assay

CPE reduction assays in Vero E6 cells were performed as previously described (28). Briefly, Vero E6 cells seeded in 96-well cell culture plates were pre-incubated with 2-fold serial dilutions of compounds for 30 min. Subsequently, cells were either mock-infected (to assess cytotoxicity of compounds) or were infected with 300 PFU of SARS-CoV-2 per well. Each well contained a total volume of 150 µL of medium with compound. Plates were incubated for 3 days at 37°C. After, cell viability was determined via a colorimetric method by measuring absorption at 495 nm with an EnVision Multilabel Plate Reader (PerkinElmer). Both EC50 (50% effective concentration, required to inhibit virus-induced cell death by 50%), and CC50 (50% cytotoxic concentration, reduces the viability of uninfected cells to 50% of control) were determined using non-linear regression with GraphPad Prism v8.0. For each compound, at least two independent experiments (each in quadruplicate) were performed.

### Virucidal Activity Assay

Compound dilutions were prepared in EMEM-2% FCS to mimic the conditions of cell-based assays. Tween-20 and Tween 40 were diluted in MilliQ water to concentrations lower than 1% as present on the composition of Lupkynis™ capsules (45). Phosphate-buffered saline (PBS) was used as a negative control and 50% ethanol as a positive control. In order to assess its virucidal activity, SARS-CoV-2 (5 × 10^4^ PFU) was incubated with the material for 2 h at 37°C with rocking. Then, serial dilutions (ranging from 10^–1^ to 10^–6^) of compound mixed with virus were prepared in EMEM-2% FCS and added to Vero E6 monolayers. After 1 h incubation, inoculum was removed and 2 ml/well of overlay containing DMEM, 1.2% Avicel (FMC BioPolymer), 2% FCS, 50 mM HEPES, and antibiotics were added. After a 3-day incubation, monolayers were fixed with 3.7% formaldehyde in PBS, and plaques were visualized using crystal violet staining (41).

### Virus Yield Reduction Assays

Calu-3 cells were seeded in 96-well plates (3 × 10^4^ cells per well). The next day, cells were pre-incubated for 60 min with 2-fold serial dilutions of CsA, TAC or VCS. Subsequently, cells were infected with SARS-CoV-2 (MOI of 1, based on titer determined on Vero E6 cells). After a 1 h incubation at 37°C, cells were washed three times with PBS and medium with compound was added. The medium was harvested at 24-h post-infection (h p.i.) and virus titers were determined by plaque assay on Vero E6 cells as described before (46). In parallel, a cytotoxicity assay with mock-infected cells treated using the same concentration of compounds was performed (*Cytopathic Effect Reduction Assay* section). VCS concentrations were measured by validated LC-MS/MS.

### Coating of Plastic Materials

The following coating solutions were prepared fresh before each experiment: BSA, 100 mg/ml bovine serum albumin (Sigma) in PBS; PEG, 1% polyethylene glycol 3350 (Sigma) in MilliQ water; Tween-40, 0.2% polysorbate 40 (Fluka) in MilliQ water; and 500 mM VCS in DMSO (Sigma). All plastic labware, including tubes and tips, was filled with each solution and incubated for 2 h at room temperature with rocking to homogenously coat the surfaces. After rinsing twice with MilliQ water, the items were left to dry at room temperature until further use in experiments.

### Virus Yield Reduction Assays in Glass Bottles

Borosilicate glass reagent bottles (50-ml) were treated with glacial acetic acid, washed twice with absolute ethanol, dried and UV-sterilized prior to use. Three times concentrated compound solutions were prepared in EMEM-2% FCS using sterile glass culture tubes, a glass syringe (Hamilton) and glass Pasteur pipettes. One ml of each compound dilution was transferred to three different reagent bottles (triplicates). Confluent monolayers of Calu-3 cells grown in culture flasks were infected with SARS-CoV-2/Leiden-002 (MOI of 1). Inoculum was removed after 1 h incubation at 37°C. Cells were washed three times with PBS, trypsinized and resuspended in EMEM-2% FCS. Two ml of this cell suspension (∼10^6^ cells) was transferred to each reagent bottle, containing compound solution. Medium was collected 24 h p.i. and virus titer was determined by plaque assay. VCS concentrations in the medium were determined by LC-MS/MS.

### Determination of Compound Cytotoxicity in Glass Culture Tubes

Calu-3 cells were trypsinized and 1.5 × 10^5^ cells in 1 ml of EMEM-2% FCS were divided over glass culture tubes. Two-fold dilutions of VCS, TAC and CsA were prepared in EMEM-2% FCS medium using glass labware, and added to corresponding tubes with cells (three tubes per concentration). After a 24 h incubation, cell viability was determined (see *Cytopathic Effect Reduction Assay* section).

## Results

### Inhibition of SARS-CoV-2 Replication in Cell Culture by Pharmaceutical Formulations of Immunosuppressive Drugs

To compare the antiviral effect of different immunosuppressive drugs commonly used in KTRs, we performed SARS-CoV-2 CPE reduction assays with VCS, cyclosporin, microemulsion (CsA_me), TAC, EVL, and MMF. These experiments were performed using the pharmaceutical formulations of the compounds to ensure optimal solubility and stability. At the start of our study only the pharmaceutical formulation of VCS) was available to us from a previous study. In each experiment, drug cytotoxicity was assessed in parallel, in non-infected cells. RDV was included as a standard positive control for inhibition of viral replication [data not shown (28)].

**FIGURE 1:**
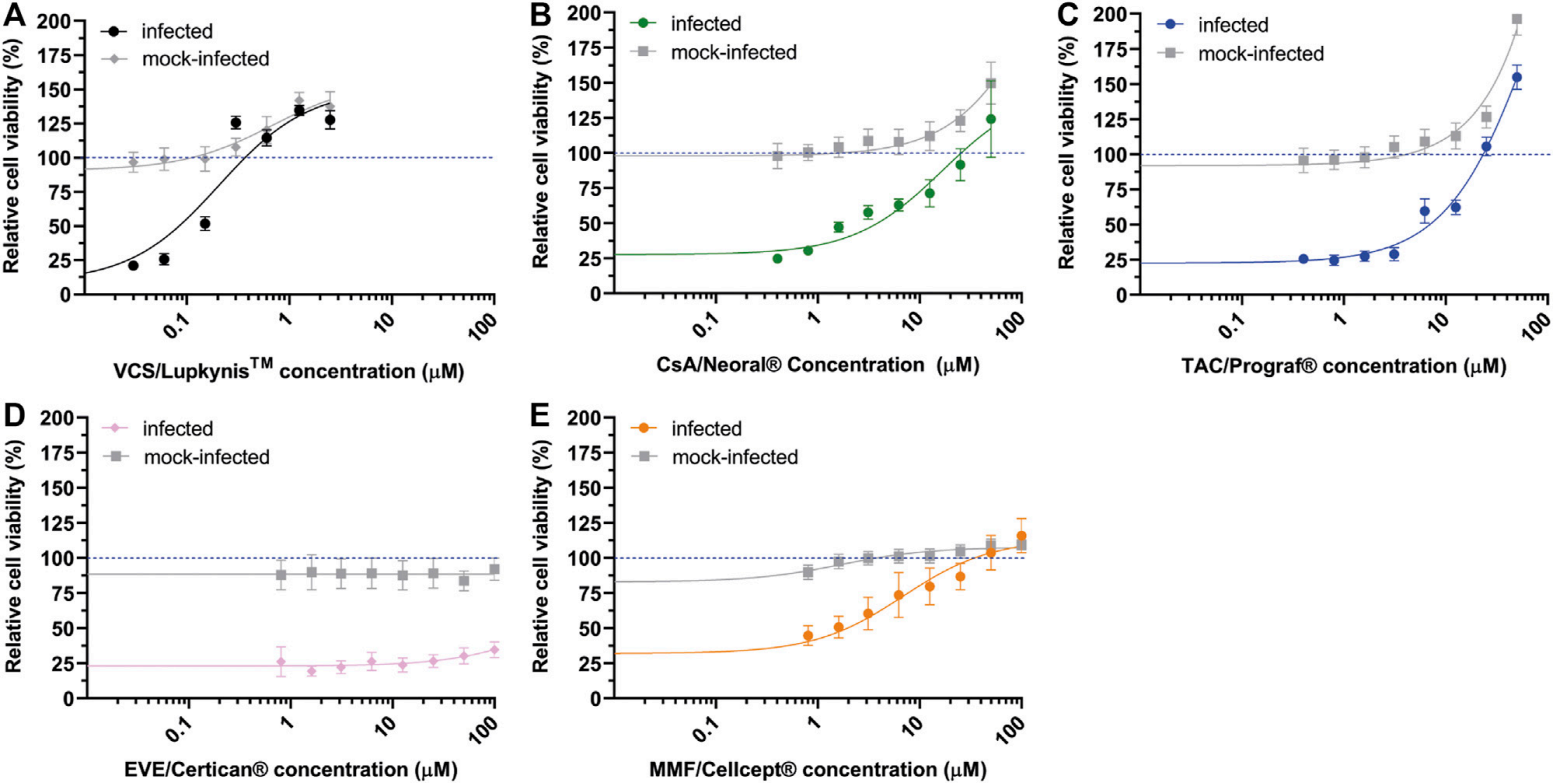
Effect of immunosuppressive drugs on SARS-CoV-2 replication. Inhibition of SARS-CoV-2 replication (colored symbols and curves) in Vero E6 cells by various drugs was determined by CPE reduction assay. For each drug, two-fold serial dilutions of the pharmaceutical formulations were tested. **(A)** VCS/Lupkynis, **(B)** CsA me/Neoral, **(C)** TAC/Prograf, **(D)** EVL/Afinitor, and **(E)** MMF/Cellcept. After preincubation with compound, Vero E6 cells were infected with SARS-CoV-2 and kept in medium containing the drug for 3 days, after which cell viability was measured with a colorimetric assay. Cytotoxicity of the drugs was evaluated in parallel using mock-infected, compound-treated cells (solid grey line). Data points represent the mean ± SD of two independent experiments. The CC50 and EC50 were determined by non-linear regression analysis and the regression curves are plotted in the graphs (solid lines).

The EC_50_ values of VCS, CsA_me and TAC were measured in the low-micromolar range, respectively: 0.22 ± 0.01 µM, 4.3 ± 0.6 µM and 10 ± 1 µM (Figures 1A–C). No inhibitory effect was observed for EVL (Figure 1D). The prodrug MMF (Figure 1E) was included in our comparison, but was not expected to inhibit virus replication, as it is likely not metabolized into its active form MPA (47) in our assay. Thus, we attributed the apparent antiviral effect of MMF to excipients present in the drug formulation (see *An Excipient in the Pharmaceutical Formulation of Voclosporin Inhibits SARS-CoV-2 Replication in Cytopathic Effect Reduction Assays* section).

Apart from VCS, none of the compounds caused cytotoxicity at tested concentrations (CC_50_ values >100 µM). Although VCS had a CC_50_ around 4 µM, its EC_50_ was also 18–45 times lower than the other compounds tested (Figure 1).

### An Excipient in the Pharmaceutical Formulation of Voclosporin Inhibits SARS-CoV-2 Replication in Cytopathic Effect Reduction Assays

**FIGURE 2:**
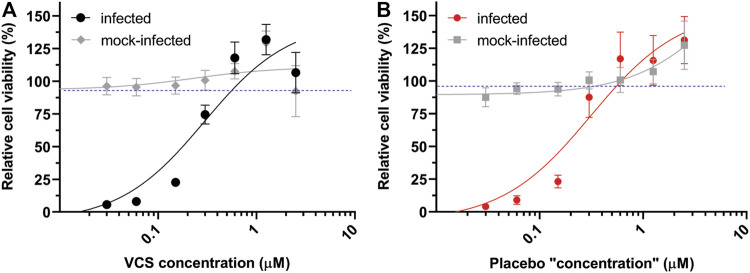
Comparison of the effect of the pharmaceutical formulation of VCS and placebo capsules on SARS-CoV-2 replication. The inhibition of SARS-CoV-2 replication in Vero E6 cells treated with the DMSO-dissolved content of VCS capsules **(A)** or placebo capsules **(B)** was determined by CPE reduction assays as described in the legend of Figure 1.

In order to evaluate whether any excipients in the pharmaceutical formulation of VCS contributed to the observed antiviral effect (Figure 1A), VCS capsules and placebo capsules were compared side-by-side using CPE reduction assay. Both capsules provided by Aurinia Pharmaceuticals Inc. had similar composition (45), with exception of the VCS compound. The absence of VCS in placebo capsules was confirmed by LC-MS/MS analysis (not shown). Surprisingly, both the VCS formulation (Figure 2A) and the placebo (Figure 2B) inhibited SARS-CoV-2 replication in a similar dose-dependent manner. This indicated that one or more excipients in the drug formulation might have an antiviral effect in this experimental setup.

**FIGURE 3:**
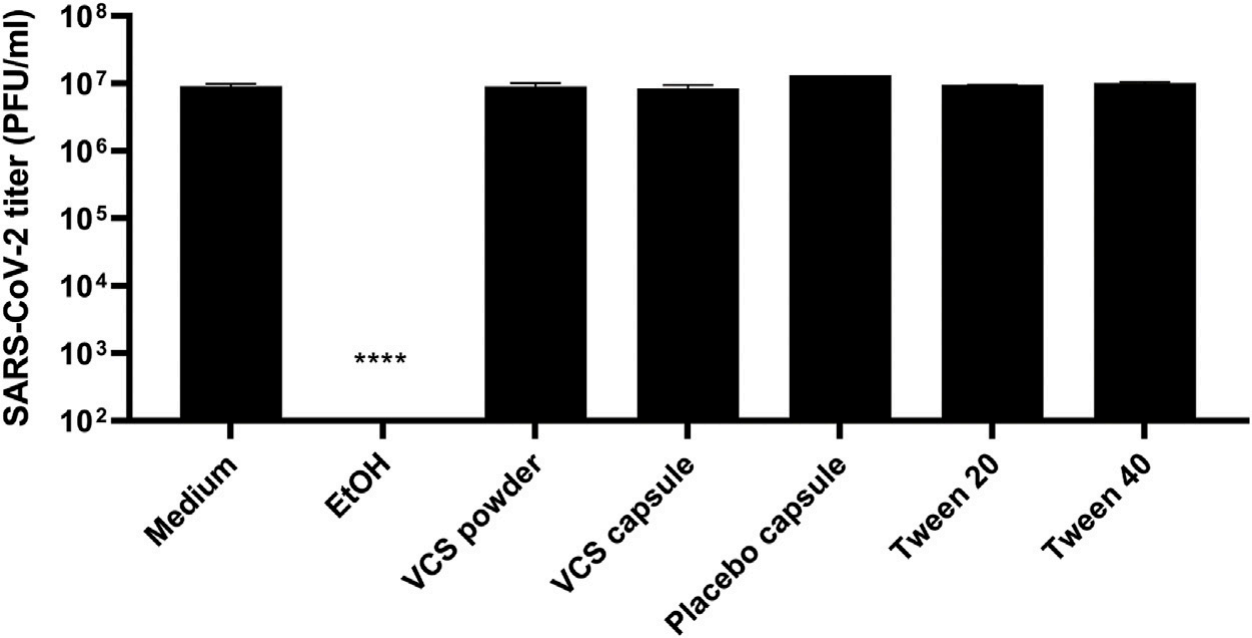
Virucidal activity of VCS and placebo capsules. The drug formulation of VCS (3.2 µM), and content of placebo capsules (corresponding to 3.2 µM VCS), and 50% ethanol were incubated with a SARS-CoV-2 virus stock for 2 h, followed by quantification of the remaining infectious virus titer by plaque assay in Vero E6. Statistical significance was determined by one-way ANOVA. *, *p < 0.1*; **, *p < 0.01*; ***, *p < 0.001*; ****, *p < 0.0001*.

Since the pharmaceutical formulation includes surfactants like Tween-20 and Tween-40 that may destroy the viral envelope, the virucidal activity of these reagents, VCS and placebo capsules were analysed. A control treatment with 50% ethanol reduced SARS-CoV-2 titers to below the limit of detection (<100 PFU/ml), while none of the other treatments significantly reduced the number of infectious particles (Figure 3). Therefore, we conclude that excipients in the drug formulation had no virucidal activity or impact on viral infectivity, but that they caused a yet poorly understood antiviral effect in the CPE reduction assays through an unknown mechanism. This invalidated the previously determined EC50 values when calcineurin inhibitors were tested using their pharmaceutical formulations (Figure 1).

### Evaluation of Antiviral Activity Using Calcineurin Inhibitors in Pure Compounds Form

**FIGURE 4:**
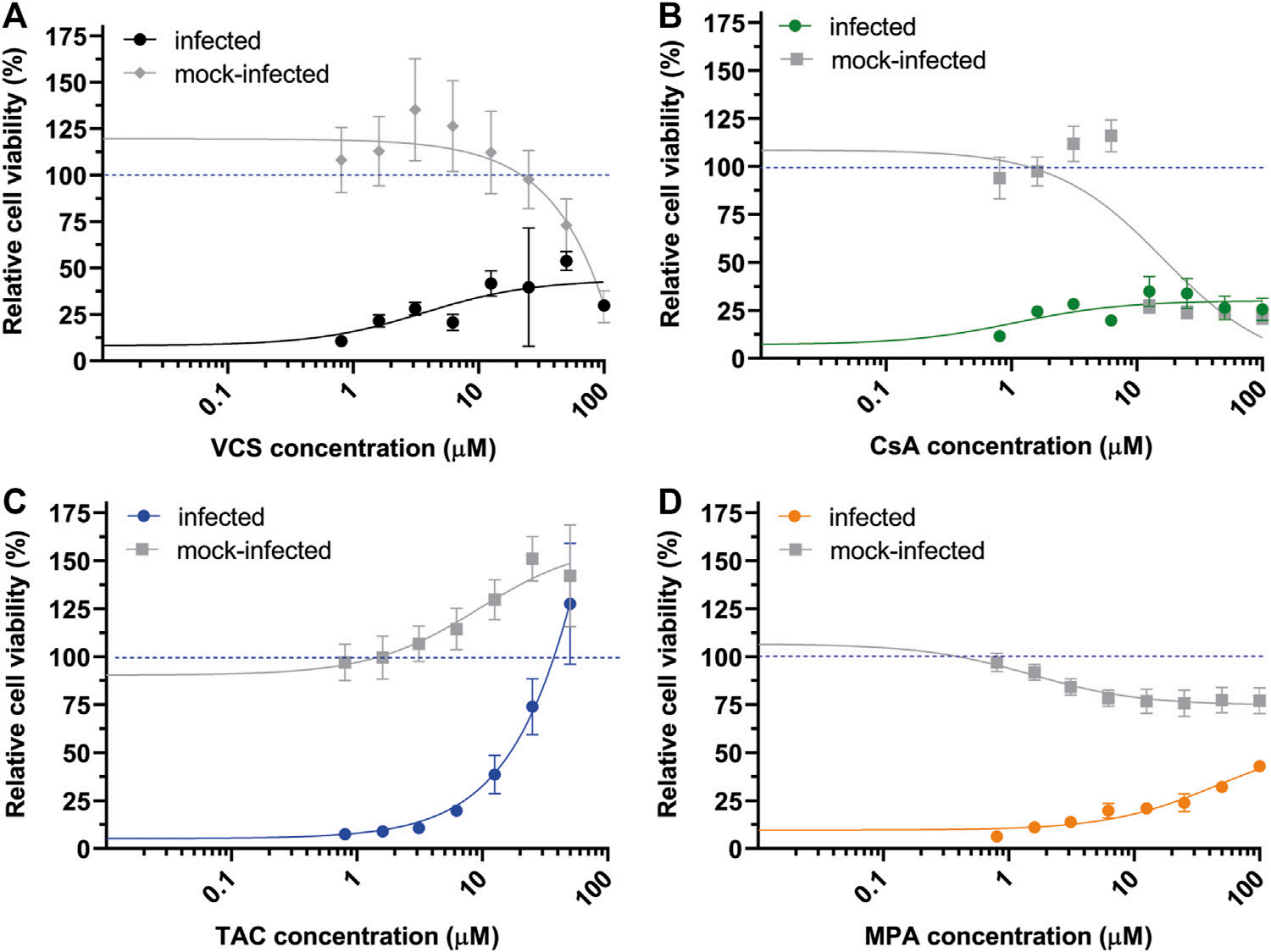
Effect of the pure active ingredients of immunosuppressive drugs on SARS-CoV-2 replication in CPE-reduction assays. **(A)** VCS, **(B)** CsA, **(C)** TAC, and **(D)** MPA. Assay was performed using Vero E6 cells. For details, see the legend to Figure 1.

To avoid interference by excipients, we performed CPE reduction assays with DMSO solutions prepared using high purity powders of the various immunosuppressive drugs. In the case of Neoral (cyclosporin microemulsion), CsA powder was evaluated. VCS solutions prepared from pure powder did not confer the same level of protection to SARS-CoV-2 infected-cells (Figure 4A) as the pharmaceutical formulation (Figure 2A). Additionally, less cytotoxicity was measured/observed CC50 > 50 µM. Similar reductions in antiviral potency were observed for CsA and MPA (Figures 4B,D), suggesting that -also in cell-based assays-these drugs need excipients to ensure solubility/bioavailability or stability for optimal activity. Interestingly, TAC solutions prepared from pure powder inhibited SARS-CoV-2 with similar efficacy as the drug formulations, i.e., with an EC_50_ of ∼15 µM (compare Figures 1C, 4C), suggesting that the pharmaceutical formulation of TAC does not contain excipients contributing to either antiviral or virucidal effects.

### Binding of Voclosporin to Plastic Strongly Affects Bioavailability and Efficacy in Antiviral Assays

We searched for potential reasons to understand the lower inhibitory effect of VCS active molecule compared to pharmaceutical formulation. Interactions between plastic and lipophilic or hydrophobic compounds, have been described (48–50). Thus, we hypothesized as VCS is highly lipophilic, it may bind to plastic which could compromise its bioavailability in standard cell-based assays using plastic labware. VCS concentrations were measured in medium using LC-MS/MS. Only 27% of the original VCS concentration could be recovered from plates due to loss of compound by binding to pipette tips and tubes during the preparation of dilutions as soon as t = 0 (Table 1).

**TABLE 1:** VCS concentration in samples incubated in plastic labware with different coatings, measured by LC-MS/MS.

Incubation time	Type of coating applied									
	Uncoated		500 mM VCS		100 mg/ml BSA solution		1% PEG-3350 solution		0.2% Tween-40 solution	
	Conc. (µM)	% remaining	Conc. (µM)	% remaining	Conc. (µM)	% remaining	Conc. (µM)	% remaining	Conc. (µM)	% remaining
0 h	0.56 ± 0.25	**28**	17.21 ± 2.36	**861**	0.55 ± 0.21	**27**	0.51 ± 0.16	**26**	0.56 ± 0.35	**28**
2 h	0.13 ± 0.07	**7**	2.73 ± 1.00	**137**	0.10 ± 0.04	**5**	0.09 ± 0.02	**4**	0.09 ± 0.04	**4**

Conc. means concentration. Note: The percentages indicate the remaining concentration relative to the concentration of the original 2 µM of VCS stock solution. The bold values indicate the percentages of VCS that remain in solution after treatment or contact.

To test whether we could prevent VCS binding to plastic in our standard antiviral assays, we coated all plastic labware using 3 different agents found in literature: BSA (51), PEG-3350 (52, 53) and Tween 40 (54). Unfortunately, none of the treatments tested was able to reduce binding of VCS to plastic (Table 1), as only 5–7% of its original concentration was recovered in solution. Alternatively, we tested if saturation of binding sites on plastic with a highly concentrated VCS solution (500 mM) prevented loss of compound. Leaching of compound from plastic was observed, resulting in unpredictable concentrations of VCS in solution, e.g., we measured a VCS concentration of >15 µM when a 2 µM stock solution was used (Table 1).

Similarly, TAC and CsA concentrations were measured using the same setup as for VCS. A 76% of the original TAC concentration and 62% of the initial CsA concentration could be recovered in solution (Table 2). This emphasized the need to use different type of materials to perform our experiments to truly evaluate these CNIs antiviral activity.

**TABLE 2:** Concentration of TAC and CsA in samples incubated in plastic labware, measured by LC-MS/MS.

Incubation time	TAC		CsA	
	Conc. in µM	% remaining	Conc. in µM	% remaining
0 h	0.85		0.76	
2 h	0.65	**76**	0.47	**62**

Note: The percentages indicate the remaining concentration relative to the concentration of the original compound stock solution (0.8 µM). The bold values indicate the percentages of VCS that remain in solution after treatment or contact.

### Inhibition of SARS-CoV-2 Replication by Voclosporin, Cyclosporin A and Tacrolimus in Calu-3 Cells

To evaluate the effect of VCS, CsA and TAC on SARS-CoV-2 replication, viral load reduction assays were performed using human lung epithelial cells (Calu-3). Moreover, we developed custom assays using exclusively glass labware to circumvent the problem of VCS binding to plastic.

**FIGURE 5:**
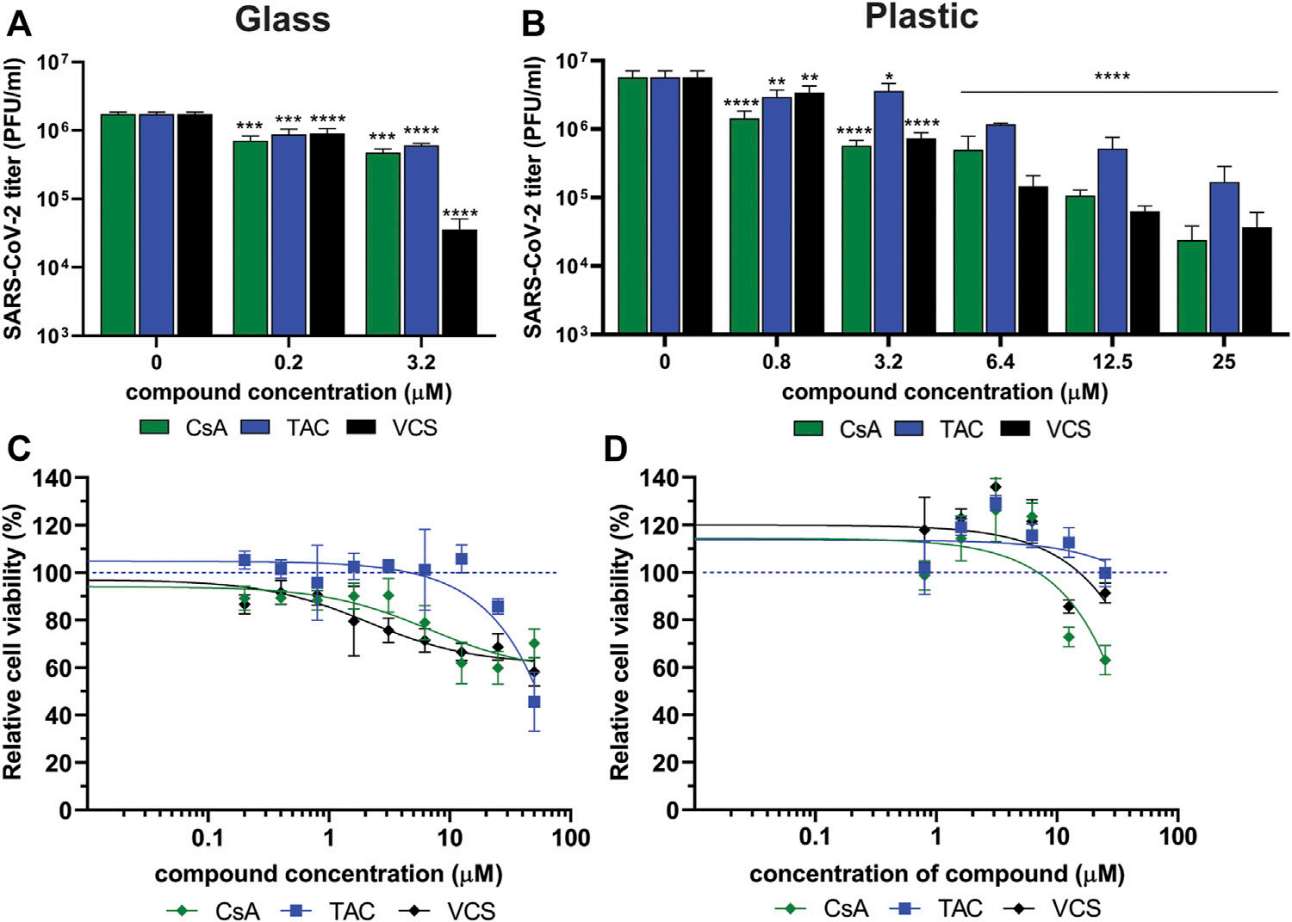
Effect of CsA, TAC and VCS treatment on the production of infectious SARS-CoV-2 progeny by human Calu-3 cells. Experiments were performed using either glass **(A,C)** or plastic labware **(B,D)**. Cells were infected with SARS-CoV-2 in the presence of different concentrations of VCS, CsA and TAC using stock solutions prepared from pure powders dissolved in DMSO. The viral load in the medium of infected cells was determined by plaque assay on Vero E6 cells using supernatant harvested at 24 h p.i. Viability of uninfected Calu-3 cells treated with the same range of compound concentrations was measured in parallel by a colorimetric viability assay (C; *n* = 12; D; *n* = 3). Mean values ±SD are shown and statistical significance of the difference between each concentration and solvent control was assessed by one-way ANOVA. *, p < 0.1; **, p < 0.01; ***, p < 0.001; ****, p < 0.0001.

Calu-3 cells in glass remained viable and supported SARS-CoV-2 replication as an increase in viral titer was measured at 24 h p.i. (Figure 5A). RDV was included as a positive control for inhibition of SARS-CoV-2 replication and assay validation. Treatment of infected cells with 10 µM RDV inhibited viral replication by > 4log(data not shown), which is in agreement with previously reported data (55). Treatment of cells with 3.2 µM VCS (pure compound) caused a more than 1.5 log reduction in SARS-CoV-2 infectious progeny titers, while an ∼0.5 log reduction was observed when the same concentration of CsA or TAC was used (Figure 5A). However, treatment with 3.2 μM VCS or CsA also caused cytotoxicity, as cell viability dropped to ∼75% (Figure 5C). Therefore, it cannot be excluded that part of the observed antiviral effect is due to pleiotropic effects (toxicity).

In experiments using plastic materials, a dose-dependent reduction in infectious progeny titers was observed when cells were treated with VCS, leading to a more than 1 log reduction at 6.4 µM (Figure 5B). CsA treatment led to a similar reduction at 25 µM. CsA displayed significant cytotoxicity at concentrations of 12.5 µM or above while VCS did not (Figure 5D). In contrast, a higher concentration of TAC (25 µM) was required to reduce the viral titer by more than 1 log. Overall, VCS showed a stronger inhibitory effect in experiments performed with glass instead of plastic labware.

Measurement of the VCS concentration in glass containers without cells demonstrated no significant loss of compound from solution (Table 3). When VCS solutions of 0.2–3.2 µM were used in glass bottles with Calu-3 cells, a ∼75% reduction of the VCS concentration was measured, suggesting the compound was bound or taken up by cells. In contrast, in experiments using standard plastic labware, we measured a 0.68 µM concentration of VCS in medium of cells treated with 25 µM VCS solution. Taking into account a similar reduction in virus titer using 3.2 and 25 µM of VCS in glass and plastic, respectively, this corroborated that when using plastic, the bioavailable amount of VCS is likely only 10% of that in the input solution.

**TABLE 3:** VCS concentration in samples from experiments using only glass labware, measured by LC-MS/MS.

Incubation time	Concentration of VCS in supplied solution											
	3.2 µM		3.2 µM		1.6 µM		0.8 µM		0.4 µM		0.2 µM	
	Without cells		With cells									
	Conc. in µM	% remaining	Conc. in µM	% remaining	Conc. in µM	% remaining	Conc. in µM	% remaining	Conc. in µM	% remaining	Conc. in µM	% remaining
0 h	2.91		2.91		1.77		0.99		0.45		0.33	
24 h	2.79	**96**	0.82	**28**	0.35	**20**	0.15	**15**	0.10	**22**	<0.07^a^	ND

a Below detection limit of LC/MS-MS. Note: The percentages indicate the ratio of the measured (true) concentration at 24 h and the concentration of the prepared solution administrated to the cells (at 0 h incubation time). The bold values indicate the percentages of VCS that remain in solution after treatment or contact.

## Discussion

Transplant recipients are at increased risk for developing a severe course of COVID-19 owing to their immunocompromised state combined with older age and comorbidities (5, 56, 57). The attributable effect of immunosuppression to a more severe course of COVID-19 and the optimal treatment is yet unclear (7, 12). As the efficacy of approved vaccines is uncertain in KTRs, it is crucial to gain more insight into the effect of immunosuppression. In this study, we evaluated the impact of VCS and different immunosuppressive compounds on the replication of SARS-CoV-2 *in vitro* using cell-based assays (Figure 1).

Previous studies demonstrated that CNIs like CsA and TAC inhibit replication of a variety of other CoVs, including SARS-CoV and Middle East respiratory syndrome (MERS) CoV (13, 20, 23–25, 58, 59). As these betacoronaviruses are closely related to SARS-CoV-2 (12, 60, 61), we expected to observe a similar inhibitory effect. In this study, we also evaluated the antiviral activity of a novel CNI, VCS (40, 62). In Calu-3 cells, VCS (pure compound) inhibited SARS-CoV-2 replication with an EC_50_ in the sub-micromolar range (<3. 2 µM), at lower concentrations than CsA or TAC (Figure 4). Our findings are in line with recent reports, showing that CsA inhibited SARS-CoV-2 replication in HuH7.5 and Calu-3 cells, but not in Vero cells (63). Notably, Dittmar et al found no activity when using TAC in any of these cell lines (63) In contrast to our finding that TAC showed antiviral activity in Vero E6 cells with an EC_50_ of ∼15 µM (Figures 1, 4). This discrepancy might be explained by use of different Vero cell subclones.

While testing the pharmaceutical formulations of CNIs, we discovered that the excipients in these preparations had (apparent) antiviral effects in our cell-based assays (Figure 2). Unexpectedly, this was not due to a virucidal effect of the surfactants in these formulations (Figure 3) which could have the potential to damage the viral envelope (64–66). This undesired effect of excipients did not allow us to proceed testing pharmaceutical formulations in our cell-based assays, as it would lead to false positive results for various compounds. This evidences the necessity of proper controls in studies investigating the potential antiviral effect of CNI’s.

VCS is a highly lipophilic compound and we observed that binding to plastic surfaces of commonly used labware strongly reduced its bioavailability in assays. We measured losses of >80% of the compound in solution (Table 1). This demonstrates that the use of plastic labware can lead to a serious underestimation of the efficacy of compounds in (antiviral) assays, in line with suggestions from previous publications (48–50). Our attempts to prevent binding of VCS to plastic by various (coating) treatments of labware were unsuccessful as none led to a more than ∼10% recovery of the initial VCS concentration (Table 1). As a solution to circumvent plastic binding, we performed experiments using glass labware, which supported growth of human Calu-3 cells and SARS-CoV-2 replication (Figure 5). Measurement of VCS concentrations by LC-MS/MS demonstrated that there was hardly any loss of the compound (Table 3). Using this setup, we demonstrated that VCS reduced the production of SARS-CoV-2 infectious progeny in a dose-dependent manner, and more effectively than CsA and TAC.

It is difficult to translate the *in vitro* finding to the clinical context. Ideally, an immunosuppressive regimen should prevent rejection, inhibit viral replication and reduce (over)inflammation, while also allowing the host to still mount an effective antiviral response. Some CNI’s are already being evaluated in clinical trials to determine their efficacy in COVID-19 patients [reviewed in (67)]. Interestingly, one study found a clear survival benefit for patients on CsA compared to other experimental anti-inflammatory therapy for COVID-19 (68). In the current study we demonstrate that cyclophilin-dependent CNIs, VCS or CsA, inhibit SARS-CoV-2 replication in cell culture more potently than TAC. VCS inhibited SARS-CoV-2 replication by ∼2log at 8-fold lower concentrations than TAC (Figure 4A). Of note, TAC concentrations that are required to inhibit SARS-CoV-2 replication likely correlate with intolerable or toxic concentrations in humans (EC_50_ of 0.2 µM equals 160 ng/ml for TAC), without even taking into account that the free fraction in traffic is only ∼10%. For CsA and VCS 0.2 µM correspond to a concentration of 241 and 243 ng/ml respectively (40, 69), which may come closer to peak concentrations *in vivo*. Moreover, the distribution of VCS over different organs might also be beneficial as concentrations in the lungs are higher than in blood (40, 45).

In conclusion, VCS reduced viral progeny yields in human Calu-3 cells at low micromolar concentrations and did so more effectively than CsA and TAC. The efficacy to prevent rejection in KTRs of VCS and TAC are considered to be comparable according to a phase 2b study (34). In cell culture, VCS inhibits SARS-CoV-2 replication at concentrations that are considered safe in humans. Therefore, VCS might be an attractive alternative CNI for therapy of patients that need calcineurin-based immunosuppression. Based solely on this study’s experimental data, we do not advocate the use of VCS merely for its potential antiviral properties. However, our data suggest a potential benefit of cyclophilin-dependent CNIs, in particular VCS. This warranted further clinical evaluation and VCS is currently under investigation in SARS-CoV-2-infected KTRs [EudraCT 2020–001467-82].

## Data Availability

The original contributions presented in the study are included in the article/supplementary material, further inquiries can be directed to the corresponding author.

## Abbreviations

CNI, calcineurin inhibitors; CoV, coronavirus; COVID-19, coronavirus disease 2019; CPE, cytopathic effect; CsA, cyclosporin A; Cyp, cyclophilin; EVL, everolimus; KTR, kidney transplant recipients; MMF, mycophenolate mofetil; MPA, mycophenolic acid; PFU, plaque-forming unit; RDV, remdesivir; SARS-CoV, severe acute respiratory syndrome coronavirus; TAC, tacrolimus; VCS, voclosporin.
